# A meta-analysis: single or double dartos flap layer in tubularized incised plate urethroplasty to prevent urethrocutaneous fistula?

**DOI:** 10.3389/fped.2023.1091242

**Published:** 2023-06-08

**Authors:** Yi Yuan, Yu-wen Wang, Yan-nei Liang, Yu-ying Wang, Jun-jie Ho, Tong-yu Peng, Zhang Zhao, Nan Deng

**Affiliations:** ^1^ Department of Urology, The Third Affiliated Hospital of Guangzhou Medical University, Guangzhou, China; ^2^ The First Clinical College, Chongqing Medical University, Chongqing, China; ^3^ Department of Pediatric Urology, Guangzhou Women and Children's Medical Center of Guangzhou Medical University, Guangzhou, China

**Keywords:** hypospadias, dartos flap, tubularized incised plate urethroplasty, fistula, meta-analysis

## Abstract

**Backgrounds:**

Urethrocutaneous fistula is one of the most common complications after urethroplasty. This meta-analysis aims to evaluate the superiority of double dartos flap to single dartos flap in preventing fistula during tubularized incised plate urethroplasty (TIPU), which is one of the most frequently used operations for hypospadias.

**Methods:**

We extracted clinical trials under the following included criteria: (1) children with TIPU; (2) a comparison of single and double flap layer; and (3) record of complications with the following excluded criteria: (1) non-comparison and (2) lack of data. Finally, 13 studies from PubMed, Cochrane Library, Scopus, and Embase have been investigated, with a total of 1,185 patients from 2005 to 2022. The quality assessment was conducted according to the Cochrane handbook and the Newcastle–Ottawa scale. A mixed-effect model was utilized to weigh the risk of fistula, phallic rotation, meatal stenosis, and wound dehiscence by the Review Manager V.5.4 software.

**Results:**

The double dartos flap layer group excels in descending the risk of postoperative fistula [odds ratio (OR) = 9.56; 95% confidence interval (CI) (4.76, 19.22); *P* < 0.00001] and phallic rotation [OR = 31.26; 95% CI (9.60, 101.84); *P* < 0.00001], while there are no differences in the rate of meatal stenosis [OR = 1.49; 95% CI (0.73, 2.70); *P* = 0.31] and wound dehiscence [OR = 2.30; 95% CI (0.80, 6.63); *P* = 0.12].

**Conclusions:**

The routine utility of a double dartos flap layer is recommended as a potential treatment during the tubularized incised plate urethroplasty.

**Systematic Review Registration:**

identifier PROSPERO CRD42022366294.

## 1. Introduction

Hypospadias is a common congenital malformation for boys, with an approximate incidence rate of 0.3% (1). The opening of the urethra can be anywhere on the penile ventral shaft, which probably has connections with the prognosis of the treatment. Surgery is regarded as an essential treatment for hypospadias, providing an excellent solution to the functional and cosmetic problems of the penis. Considering the children's physical and psychological development, it is known that the best timing for the operation is between 6 months and 18 months (2). Tubularized incised plate urethroplasty (TIPU) is currently one of the most popular treatments for hypospadias repair. Postoperative complications include urethrocutaneous fistula (UCF), meatal stenosis, and skin necrosis. UCF is found with the most morbidity of 5%–23% (3). To reduce the incidence of complications, Kamal in 2005 (4) introduced a method of covering the neourethra with a double layer of dartos tissue. Previous studies have confirmed its remarkable results (5, 6). However, only a few indicated that the difference between single or double dartos layers is statistically insignificant (7).

Therefore, we conducted a meta-analysis of studies comparing single and double dartos flap (DDF) layers in TIPU to evaluate whether DDF is a more appropriate treatment to reduce postoperative complications.

## 2. Materials and methods

### 2.1. Information sources

Two authors independently searched the databases, including PubMed, Embase, Scopus, and Cochrane Library, using both Mesh words and Entry Terms to select the relevant articles for our inclusion criteria by 9 October 2022. Ultimately, one investigator checked the included studies as a final revision. The Mesh words have the following: “children” and “hypospadias”, and the Entry Terms included “double”, “single”, and “dartos”.

### 2.2. Inclusion and exclusion criteria

The inclusion criteria included the following:

(1) Study design: randomized controlled trials (RCTs) and retrospective studies completed in any country or region
(2) Patients: boys aging from 9 months to 12 years, with hypospadias, need a distal penile hypospadias repair, and without preoperative topical androgen therapy
(3) Intervening measure: double dartos flap layer in tubularized incised plate urethroplasty or single dartos flap (SDF) layer in tubularized incised plate urethroplasty
(4) Primary outcomes: postoperative complications (fistula rate, bleeding or hematoma, wound infection, meatal stenosis, and phallic rotation), skin necrosis, the satisfaction of patients or their parents, and the situation of postoperative micturition

The exclusion criteria included the following:

(1) Clinical trials without the comparison of single and double dartos flap layer, case reports with seldom data, review articles, empirical perspectives, conference articles, and studies without objective data
(2) The efficacy of the risk factors related to hypospadias is unable to be determined from the trials.

### 2.3. Data collection

Two reviewers worked on the data collection procedure all by themselves. The necessary data and information were extracted and organized into tables using Microsoft Excel. The following data and information were extracted:

(1) Surgical features: subtype of hypospadias, ventral curvature, previous repair, duration of surgery, follow-up time, previous circumcision, and uncircumcised
(2) Surgical outcome: bleeding or hematoma, skin necrosis, meatal stenosis, wound dehiscence, phallic rotation, and UCF
(3) Metabolic parameter: body weight
(4) Other variables: participant, country, and age

If there were any discrepancies in the data or disagreements between the two investigators, the third-party investigator would check the study and decide on the general policy after proper discussion.

### 2.4. Quality assessment

Two researchers evaluated the quality of the RCTs following the Cochrane handbook. The Cochrane handbook concentrates on assessing the risk of bias, and the selection bias, performance bias, detection bias, attrition bias, and reporting bias are included, with the level of low risk, unclear risk, and high risk on each item of bias assessment. The Cochrane risk of bias tool was utilized with the Review Manager V.5.4 software.

The Newcastle–Ottawa scale (NOS) was utilized to assess the quality of retrospective studies. Each item of the NOS assessment is scored between 1 and 18. A retrospective trial or case-control study that scored 7 or more was deemed a high-quality trial.

If dissenting opinions occur during the quality assessment between the two investigators, the disputed study or data would be sent to the third-party investigator to decide the final results.

### 2.5. Data analysis

In this process, we analyzed data from selected studies using the Review Manager V.5.4 software. The odds ratios (OR) and 95% confidence interval (CI) were calculated and visualized with the forest plot in the Review Manager V.5.4. Secondly, we accessed the mean value and SD from continuous results. Ultimately, we measured these outcomes by using a random-effects model. The heterogeneity of every statistical test could be seen from the *I*^2^ value. We need to consider the following explanations: 0%–40% implied low heterogeneity, 50%–70% exhibited medium heterogeneity, while >70% means extremely high heterogeneity.

In addition, to reduce the heterogeneity of primary outcomes, it is necessary to conduct a sensitivity analysis with the method of excluding literature, and a subgroup analysis is conducted when the clinical characteristics are complete in every included study. Moreover, Egger's test was applied to evaluate the publication bias of each primary outcome. The two-tailed *P*-value <0.05 was deemed statistically significant, which indicated a positive result in the primary outcome. Besides, this meta-analysis abides by the PRISMA guidelines and the AMSTAR checklist for meta-analysis and systematic review.

## 3. Results

### 3.1. Study selection

Initially, a total of 329 studies were selected from PubMed (*n* = 19), Embase (*n* = 77), Cochrane Library (*n* = 78), and Scopus (*n* = 155). A total of 255 studies remained after removing duplicate allusions. During the first selection process, through titles and abstracts in each study, 197 articles were deleted, including case reports (*n* = 49), irrelevant interventions (*n* = 104), lack of comparisons (*n* = 18), and review articles (*n* = 26). Of the remaining 58 articles, 41 were removed during the second selection process, including irrelevant interventions (*n* = 41) and review articles (*n* = 4). Ultimately, the 13 most relevant clinical trials were retained as valuable data (Supplementary Figure S1) (4, 6–17).

### 3.2. Study characteristics

In the 13 studies from 1997 to December 2020, a total of 1,185 patients with hypospadias were in the SDF group (*n* = 547) and the DDF group (*n* = 638). Four randomized controlled trials and a retrospective cohort study were included, and the others were case-control studies. Most of the operations of the studies used dorsal dartos flaps, only one used ventral, and one was unclear. Moreover, the sizes of the catheter were mainly 6–10 F. The mean age varied from 12 months to 87.6 months, and the follow-up time ranged from 2.1 months to 11.25 months. Furthermore, the distal shaft hypospadias was the most common (*n* = 194). In the SDF group, the most common hypospadias subtype was coronal (*n* = 129), and in the DDF group was distal shaft (*n* = 109) (Tables 1, 2).

**Table 1:** Main characteristics of included studies.

Study	Year	Country	Time range	Study design	Dorsal or ventral	Catheter	Sample size			Mean age (month)		Follow-up time (month)	
							Total	SDF	DDF	SDF	DDF	SDF	DDF
Al-Taher (8)	2022	Asia–Jordan	January 2017–December 2020	Retrospective cohort study	Dorsal	Suitably sized catheter	163	116	47	14 ± 8.9	20 ± 8.9	12 ± 0	12 ± 0
Appignani (9)	2009	Europe–Italy	October 2002–March 2008	Case-control study	Dorsal	6–8F	97	40	57	48 ± 28.68	39 ± 27	54 ± 0	26 ± 0
Bakan (10)	2007	Asia–Turkey	2002–2006	Case-control study	Dorsal	6–10F	74	29	45	87.6 ± 45	87.6 ± 45	21 ± 0	21 ± 0
Cimador (11)	2013	Europe–Italy	January 2008–December 2011	Randomized cotrolled trial	Ventral	8F	73	36	37	21 ± 5	21 ± 5	18 ± 10.5	18 ± 10.5
Elsayed (12)	2011	Asia–Arab	January 2008–December 2011	Case-control study	Dorsal	6–8F	59	32	27	46.8 ± 15.6	43.2 ± 13.2	12.2 ± 3.8	12.2 ± 3.8
Erol (7)	2009	Asia–Turkey	2008–December 2009	Randomized cotrolled trial	Dorsal	6–8F	77	37	40	29.4 ± 17.4	28.8 ± 15.9	34 ± 0	34 ± 0
Kamal (4)	2005	Asia–Saudi Arabia	March 2002–December 2004	Randomized cotrol trial	Dorsal	8F	96	54	42	—	—	6 ± 2.75	6 ± 2.75
Maarouf (13)	2012	African–Egypt	February 2009–June 2011	Case-control study	Dorsal	6–8F	100	52	48	14 ± 4.25	12 ± 4	14 ± 4.25	12 ± 4
Naumeri (14)	2021	South Asia–PAK	August 2017–February 2018	Randomized cotrolled trial	Dorsal	Appropriate nelaton catheter	60	30	30	73.2 ± 49.2	93.6 ± 40.8	—	—
Safwat (15)	2012	Africa–Egypt	May 2005–October 2010	Case-control study	Dorsal	6F	58	28	30	41.5 ± 21	41.5 ± 21	8.6 ± 5.8	8.6 ± 5.8
Suoub (16)	2013	—	January 2008–month 2011	Case-control study	—	Stent insertion	94	44	50	16 ± 21.5	16 ± 21.5	18.2 ± 6.5	18.2 ± 6.5
Yiğiter (6)	2010	Asia–Turkey	April 2002–July 2009	Case-control study	Dorsal	—	155	23	132	90 ± 52.8	78 ± 48	8.3 ± 2.1	11 ± 6.6
Yildiz (17)	2010	Asia–Turkey	1997–2008	Case-control study	Dorsal	6–10F	79	26	53	87.6 ± 43.5	87.6 ± 43.5	28 ± 11.25	28 ± 11.25

**Table 2:** The subtypes of hypospadias in the included studies.

Study	Year	Glandular		Cornal		Subcoral		Distal shaft		Mid-shaft		Proximal	
		SDF	DDF	SDF	DDF	SDF	DDF	SDF	DDF	SDF	DDF	SDF	DDF
Al-Taher (8)	2022	25	12	86	23	0	0	2	10	3	2	0	0
Appignani (9)	2009	0	0	0	0	20	26	16	24	4	7	0	0
Bakan (10)	2007	0	0	0	0	0	0	25	28	4	14	0	3
Cimador (11)	2013	—	—	—	—	—	—	—	—	—	—	—	—
Elsayed (12)	2011	0	0	0	0	0	0	21	20	4	6	7	1
Erol (7)	2009	0	0	24	25	13	25	0	0	0	0	0	0
Kamal (4)	2005	—	—	—	—	—	—	—	—	—	—	—	—
Maarouf (13)	2012	—	—	—	—	—	—	—	—	—	—	—	—
Naumeri (14)	2021	0	0	19	6	9	9	21	27	9	3	0	0
Safwat (15)	2012	—	—	—	—	—	—	—	—	—	—	—	—
Suoub (16)	2013	—	—	—	—	—	—	—	—	—	—	—	—
Yiğiter (6)	2010	—	—	—	—	—	—	—	—	—	—	—	—
Yildiz (17)	2010	—	—	—	—	—	—	—	—	—	—	—	—

### 3.3. Quality assessment

Following the articles evaluated by the Cochrane risk tools, four studies were at low risk of bias in the selection bias, three articles were at low risk in the allocation concealment selection bias, and only one study was at unclear risk. Regarding the risk of performance bias, three trials were deemed low risk, and one article was considered unclear risk. Four trials were considered low risk in the detection bias, and four articles were estimated as low risk in the attrition bias. In addition, the risk of reporting bias and other biases were assessed as low risk in all the included studies (Supplementary Figure S2).

Each item of the NOS assessment ranged from 1 to 18, and trials that scored seven or more were deemed high quality. Of note, seven trials scored 7 points or more that were assessed as high quality, while one study scored 6 points, and the other was 4 points, which were considered as low quality in this meta-analysis. In our included studies, only Al-Taher's study was a cohort study, and eight were case-control studies. Al-Taher's study did not show the comparability of cohorts based on the design or analysis. All the case-control studies had no selection of controls, which may cause information bias. Four case-control studies did not show the definition of controls. Suoub's study did not reflect the ascertainment of exposure and the same method for cases and controls (Supplementary Table S1).

### 3.4. Primary outcomes

#### 3.4.1. Meta-analysis of UCF

**Figure 1:**
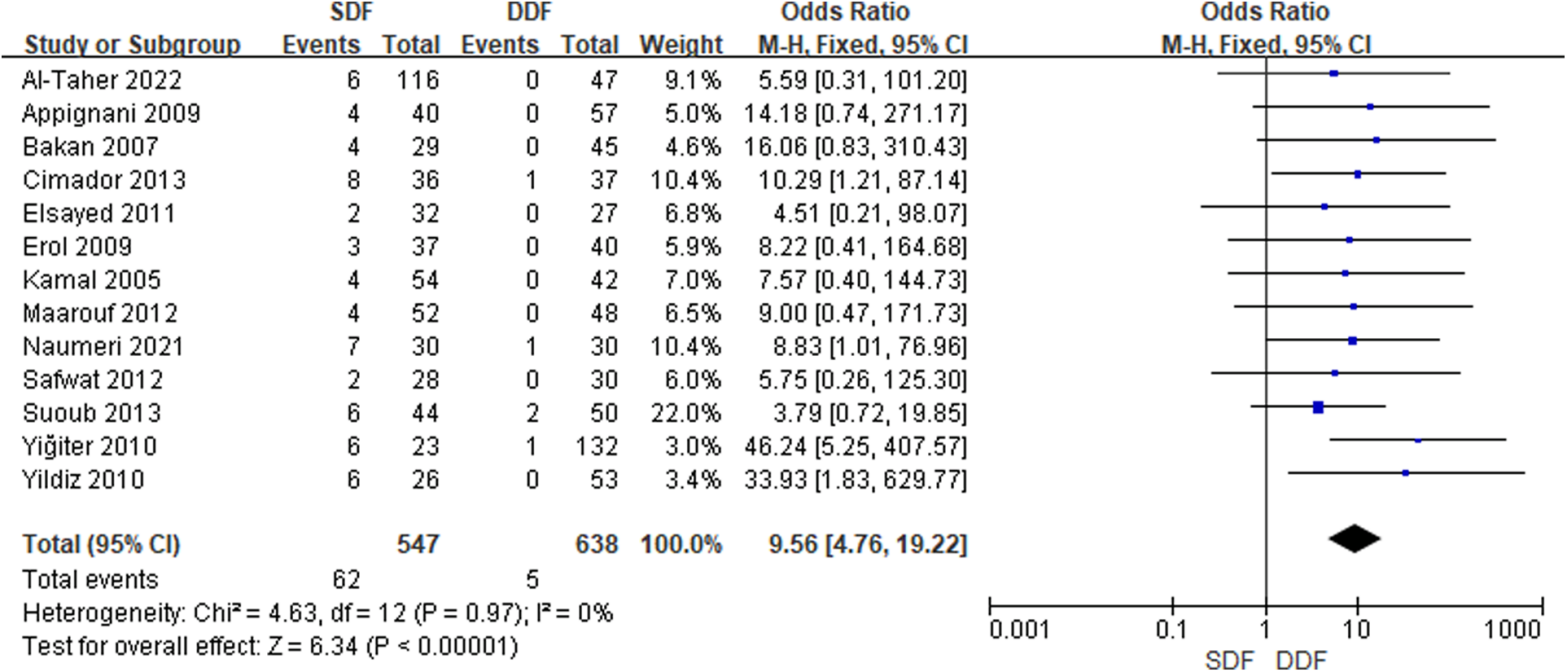
Forest plot of the risk of UCF.

A total of 13 studies reporting the results of the occurrence of UCF included 365 boys. An overt statistical significance was calculated in the occurrence relative to baseline between the SDF and DDF [OR = 9.56; 95% CI (4.76, 19.22); *P* < 0.00001]; heterogeneity was low (*I*^2^ = 0%). There was no publication bias detected after the Egger's test (Figure 1).

#### 3.4.2. Meta-analysis of meatal stenosis

**Figure 2:**
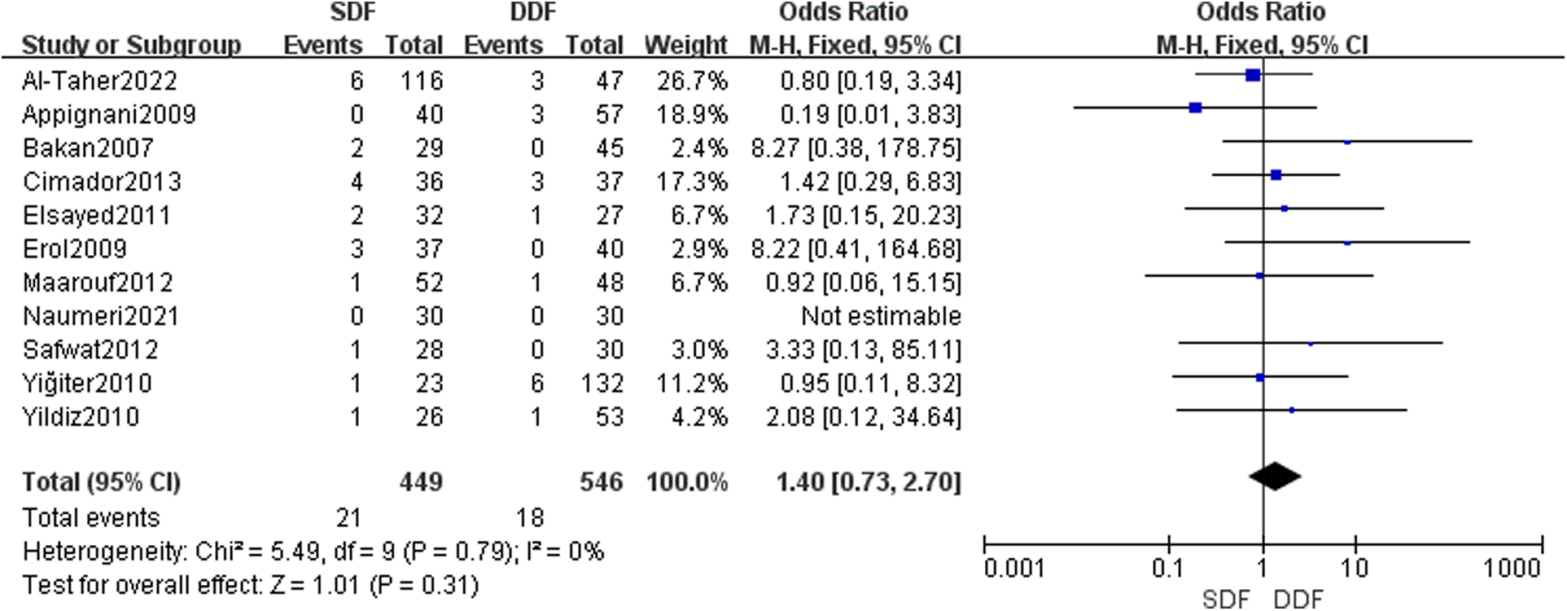
Forest plot of the risk of meatal stenosis.

Based on the meta-analysis of meatal stenosis between the SDF and DDF group that included 995 patients, there were 11 studies concerning the result of meatal stenosis after the surgery. The analysis concluded that there was no enormous difference between the SDF group and the DDF group [OR = 1.49; 95% CI (0.73, 2.70); *P* = 0.31], with low heterogeneity (*I*^2^ = 0%). There was no publication bias detected after the Egger's test (Figure 2).

#### 3.4.3. Meta-analysis of wound dehiscence

**Figure 3:**
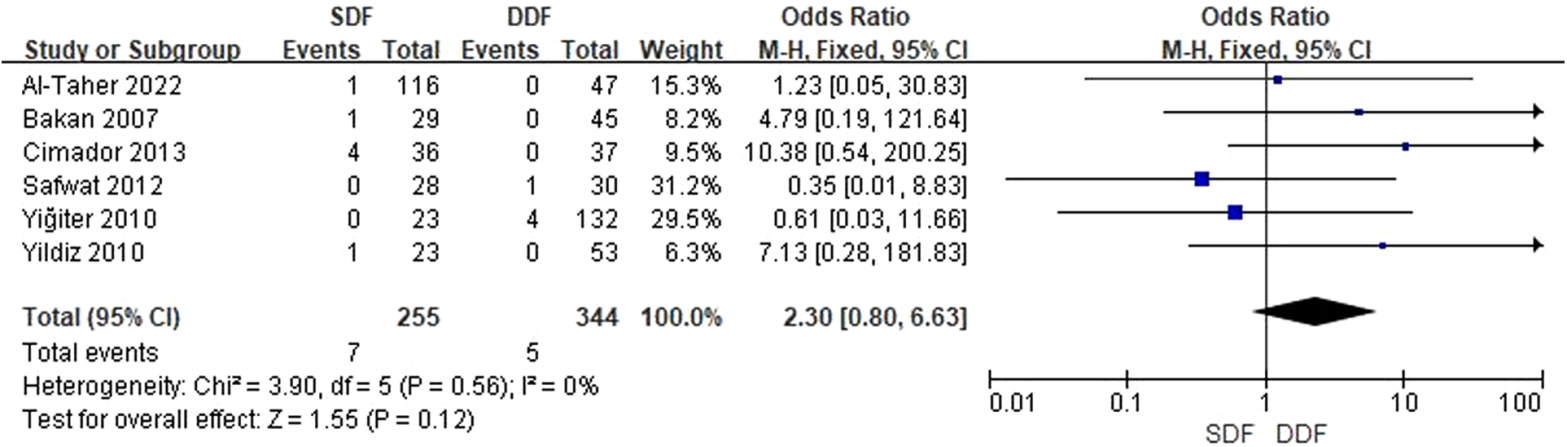
Forest plot of the risk of wound dehiscence.

Six studies reporting the occurrence of wound dehiscence relative to baseline included 255 patients. The pooled analysis showed that there was no difference in the occurrence of wound dehiscence relative to baseline between the SDF and DDF [OR = 2.30; 95% CI (0.80, 6.63); *P* = 0.12], with low heterogeneity (*I*^2^ = 0%). In addition, the publication is unbiased, which was assessed by the Egger's test (Figure 3).

#### 3.4.4. Meta-analysis of phallic rotation

**Figure 4:**
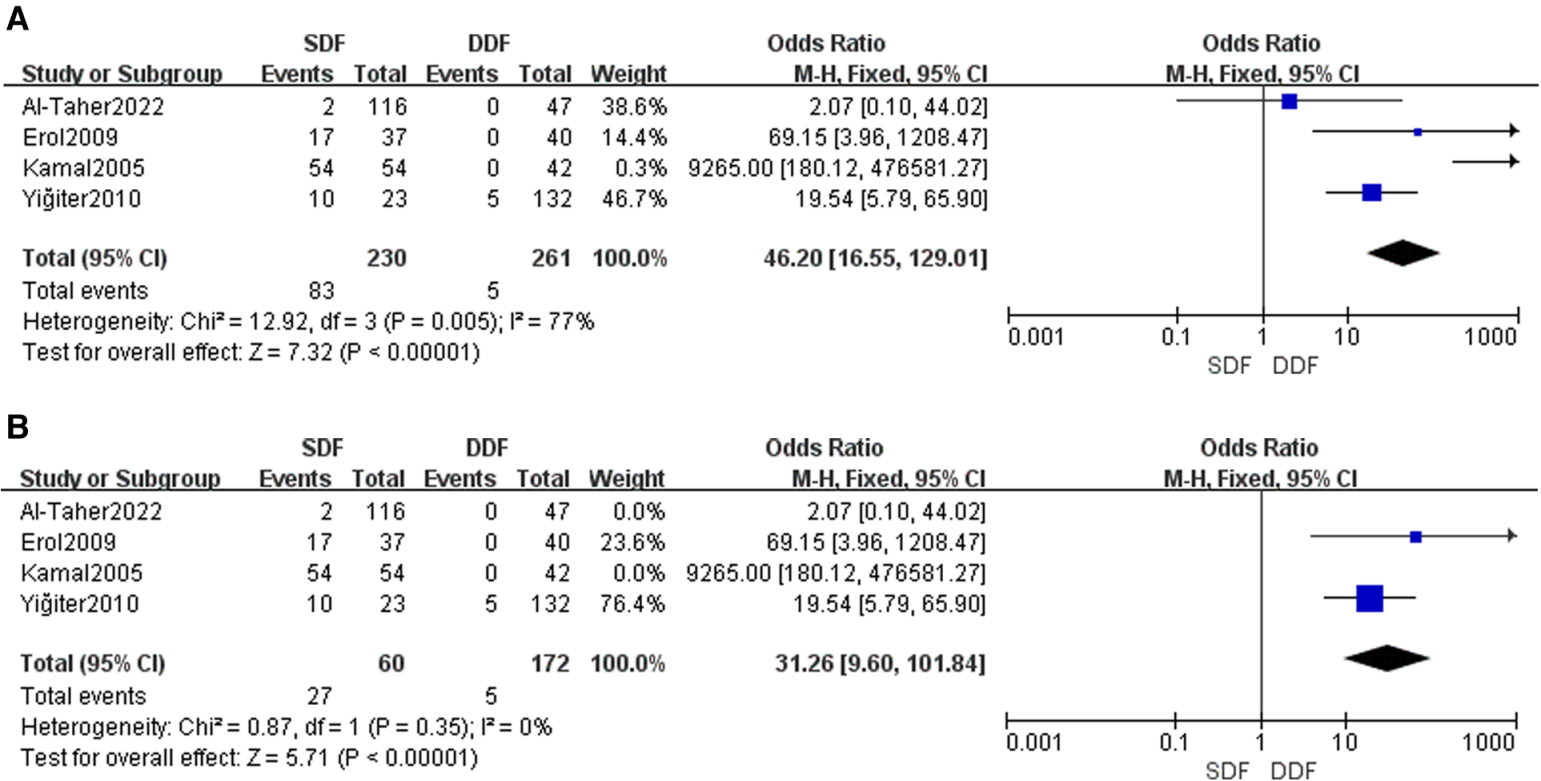
Forest plot of the risk of phallic rotation. (**A**) The risk of phallic rotation with high heterogeneity. (**B**) The risk of phallic rotation with low heterogeneity.

The meta-analysis of phallic rotation between the SDF group and DDF group, including 491 patients, revealed four studies concerning the result of phallic rotation after the surgery. The analysis concluded that there was an enormous difference between the SDF group and the DDF group [OR = 46.20; 95% CI (16.55, 129.01); *P* < 0.00001], with high heterogeneity (*I*^2^ = 77%). Compared to alternative studies, the article written by Kamal in 2005 and the article written by Al-Taher in 2022 were the roots that led to high heterogeneity. After deleting Kamal's and Al-Taher's studies, the total heterogeneity declined to 0%. Meanwhile, the comprehensive effect of the analysis showed a manifest distinction between the SDF group and the DDF group [OR = 31.26; 95% CI (9.60, 101.84); *P* < 0.00001]. In addition, the publication is unbiased, which was assessed by the Egger's test (Figure 4).

## 4. Discussion

A de-epithelialized preputial dartos flap has been implemented frequently since Retik et al. initially reported the use of preputial dartos for neourethra covering (18). Notably, when flap production was asymmetrical or insufficient, Retik's description of lateral transposition served as a limiting factor. Gonzalez first reported and adopted the idea of creating a buttonhole on the flap, allowing the penis to pass through (19), which was named TIPU or the Snodgrass surgery. TIPU then acquired widespread recognition for improving cosmetic and functional outcomes, and it is increasingly popular for proximal hypospadias (5, 20–22). Nevertheless, there are still multiple postoperative complications such as urethral stricture, meatal stenosis, wound dehiscence, and UCF (23, 24). Kamal initiated a surgical procedure of a double dartos flap covering on the neourethra to prevent UCF (4). Of note, the comparison of single and double dartos flap remained unknown. This meta-analysis aims to evaluate their efficacy in preventing UCF.

A total of 13 clinical trials with 1,185 patients suffering from different types of hypospadias were included in this meta-analysis. Seven retrospective cohort studies and case-control studies were evaluated with the NOS scale, and four randomized controlled trials were assessed according to the Cochrane handbook.

As numerous studies have demonstrated, this meta-analysis study revealed that the use of a double dartos flap layer during urethroplasty was able to lessen fistula formation in cases of distal penile hypospadias. In terms of lower fistula rates after the double dartos flap, we assumed that the second dartos flap and penile skin tissue would protect the small perforation from becoming a fistula. Moreover, a fistula track can form *via* the suture line right near the flap edge when only one dartos flap is implemented. In contrast, with double dartos flaps, two pedicles are rotated from both sides and fixed across the neourethra, which increased safety in case of the emergence of fistulas by sealing the neourethra from the glans and skin on the front as well as on both sides (4). Furthermore, the covering of a three-layer tissue (two dartos flap layers and penile skin) provides a more effective tissue supplement to the neourethra and sufficient supplies of blood flow. This new finding facilitates urethral recovery by improving the nutrition of the adjacent neourethra (18). Nowadays, studies reporting DDF on proximal hypospadias are scant to be able to draw conclusions, and more clinical trials in this aspect are needed. Elsayed pointed out that seven patients in the SDF group and one patient in the DDF group had proximal hypospadias. In the aspect of complications, Elsayed found that two patients in the SDF group had a urethral fistula, while no patient in the DDF group had a urethral fistula. Bakan's research only found one patient in the DDF group with proximal hypospadias, and no urethral fistula was reported in the DDF group. Overall, DDF may have more advantages than SDF in the treatment of distal and proximal hypospadias, but further clinical trials are required to confirm the hypothesis.

The results showed that there is no statistically significant difference in the incidence of meatal stenosis between the SDF and the DDF groups, which were consistent with Snodgrass's study that there was no direct connection between the incidence rate of meatal stenosis and the choice of single or double dartos. Snodgrass supposed that doctors probably failed to incise the plate deeply enough or sewed the plate too far distally, thus inducing meatal stenosis (17). Notably, Elbakry reckoned that the double flap layer was a risk factor for meatal stenosis due to urethral compression (4). Further studies are required to explore the association between meatal stenosis and the use of a double flap layer in TIPU.

With respect to the other complications, like wound dehiscence analyzed in our study, there was no difference between the SDF and the DDF group. We supposed that it is an unavoidable postoperative complication of surgery, which might be related to surgeon expertise. Although there was no statistical difference in the data analysis, we could still find that the rate of wound dehiscence was lower in the DDF group than that in the SDF group. In the SDF group, it is impossible to transpose the flap to the ventral aspect of the penis, which can result to deteriorated vascular supply. In contrast, DDF is long enough to cover the neourethra with additional protection for the vascular supply. Therefore, further clinical trials are also needed to investigate the corresponding mechanism. To summarize, using a double dartos flap may prevent and reduce the occurrence only to a certain extent (25).

A statistically significant difference in the incidence of phallic rotation was found. Notably, the DDF group had a lower risk of penile rotation, regardless of the heterogeneity. Blood supply and fascia situation are investigated to find out when it comes to the reason. Erol suggested that phallic rotation was related to the regular blood supply of the flap in the single-layer dartos flap repair procedure (7). Simultaneously, Yiğiter insisted that if the ventral fascia is insufficient, the dartos flap used for urethral formation is insufficient, and the flap asymmetry may lead to phallic rotation (6). Yiğiter and Erol mentioned a slight rotation during the procedure when the dartos flap on one side was sutured. The phallic rotation disappeared when the flap on the other side was sutured in the opposite direction (4, 6).

There are several limitations in this meta-analysis. First, due to several retrospective studies, the study with selection bias and heterogeneity is of note. Second, the preputial vascular anatomy varied from study to study. The morphology and blood supply of the prepuce are crucial for hypospadias repair, while some studies failed to emphasize the well-vascularized dartos flap. Moreover, when the preputial dartos flap is divided into right and left halves, it is not guaranteed that both flaps have a similarly good blood supply (26). Finally, due to the limited number of cases, only four complications were analyzed in our research. Other noteworthy outcomes concerning cosmetic results and skin necrosis are neglected.

## 5. Conclusion

Based on this meta-analysis, the covering of the double dartos flap layer enables a lower risk of UCF after the TIPU. Simultaneously, phallic rotation is avoidable with double dartos flap layers during the TIPU. Notably, the use of a double flap layer could reduce the dehiscence rate. We recommend the routine utility of a double dartos flap during the tubularized incised plate urethroplasty. Further clinical trials should be conducted to illustrate the association among the double flap layer, meatal stenosis, and blood supply.

## Data Availability

The original contributions presented in the study are included in the article/**Supplementary Material**; further inquiries can be directed to the corresponding author.
