# Supplementary figures and images for: A meta-analysis: single or double dartos flap layer in tubularized incised plate urethroplasty to prevent urethrocutaneous fistula?
Source: Front Pediatr. 2023 Jun 8;11:1091242. doi: 10.3389/fped.2023.1091242 (PMC10286861; doi:10.3389/fped.2023.1091242)

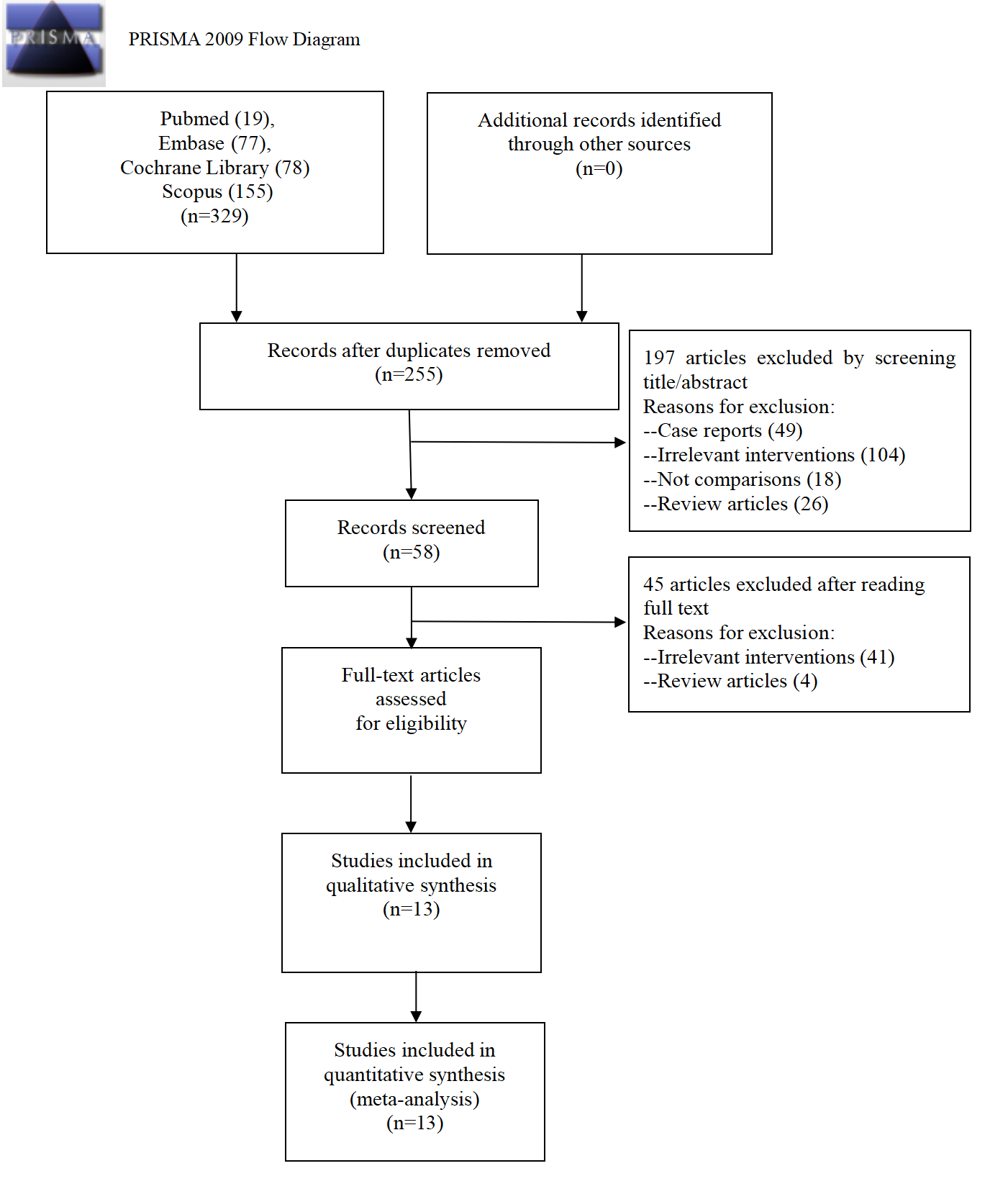

Supplement: Supplementary file 1 [file Image1.png]

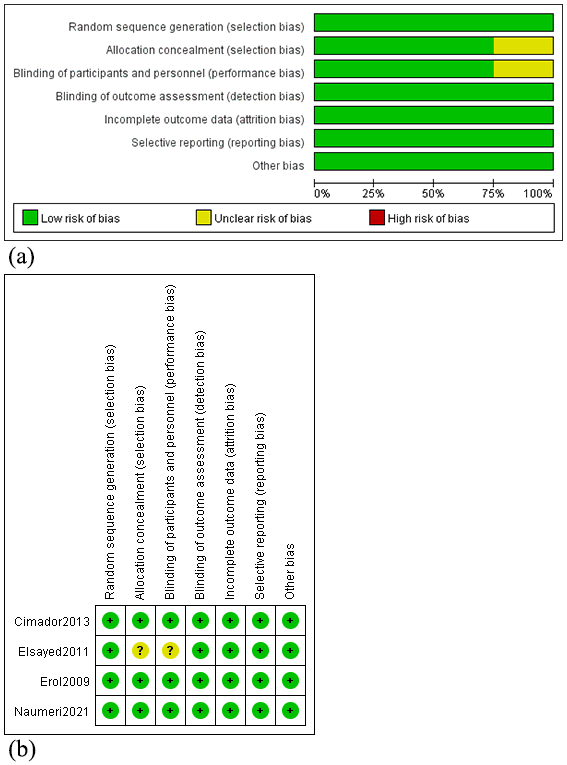

Supplement: Supplementary file 2 [file Image2.png]

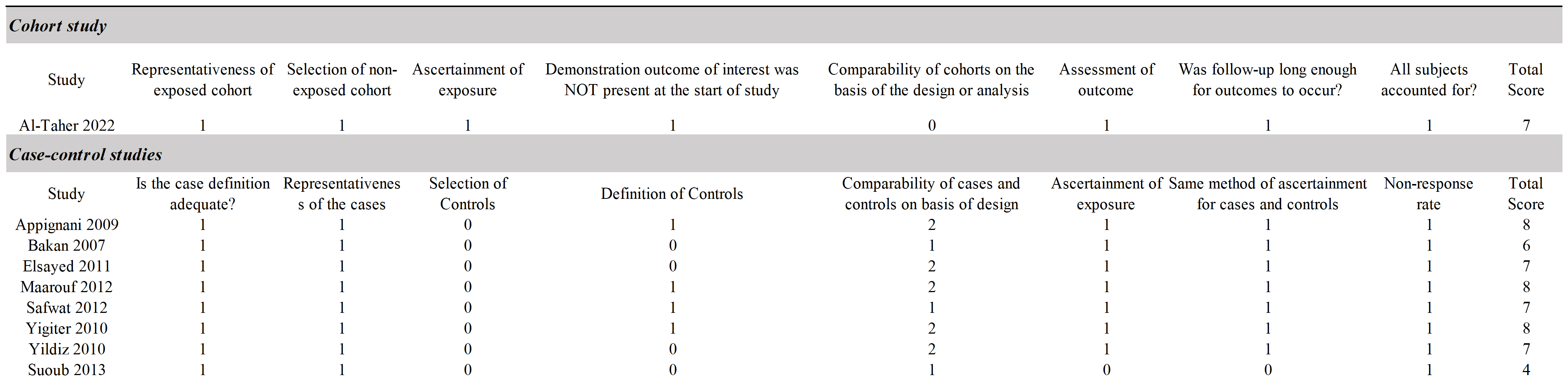

Supplement: Supplementary file 3 [file Image3.png]
